# Dynamic Responses of Rhizosphere Microorganisms to Biogas Slurry Combined with Chemical Fertilizer Application during the Whole Life Cycle of Rice Growth

**DOI:** 10.3390/microorganisms11071755

**Published:** 2023-07-05

**Authors:** Zhenbao Shi, Yanmei Yang, Yehong Fan, Yan He, Tian Li

**Affiliations:** 1 State Key Laboratory of Pollution Control and Resource Reuse, Tongji University, Shanghai 200092, China; shizhenbao2@163.com (Z.S.); tianli@tongji.edu.cn (T.L.); 2 Shanghai Engineering Research Center of Biotransformation of Organic Solid Waste, Shanghai Key Laboratory for Urban Ecological Processes and Eco-Restoration, School of Ecological and Environmental Sciences, Institute of Eco-Chongming, East China Normal University, Shanghai 200241, China; yanmeiyangecnu@163.com (Y.Y.); fanyh980718@163.com (Y.F.)

**Keywords:** biogas slurry plus chemical fertilizer, rhizosphere microorganisms, paddy fields, bacteria, fungi

## Abstract

Biogas slurry combined with chemical fertilizer (BCF) is widely used as a fertilizer in paddy fields and rhizosphere microorganisms are key players in plant growth and reproduction. However, the dynamic responses of rhizosphere microorganisms of field-grown rice to BCF application still remain largely unknown. In this study, a field experiment was conducted in two proximate paddy fields in Chongming Island to study the impacts of BCF on the changes in rhizosphere microorganisms during the whole rice growth, including seedling, tillering, booting, and grain-filling stages, with solely chemical fertilizer (CF) treatment as control. The results showed BCF could increase the N-, P-, and C- levels in paddy water as well as the rhizosphere microbial abundance and diversity compared with control. In particular, the phosphate-solubilizing- and cellulose-decomposing-bacteria (e.g., *Bacillus*) and fungi (e.g., *Mortierella*) were more abundant in the rhizosphere of BCF than those of CF. Moreover, these microbes increased markedly at the booting and grain-filling stages in BCF, which could promote rice to obtain available nutrients (P and C). It was noted that denitrifying-like bacteria (e.g., *Steroidobacteraceae*) decreased and dissimilatory nitrate reduction to ammonia-related bacteria (e.g., *Geobacter*, *Anaeromyxobacter*, and *Ignavibacterium*) increased at the booting and filling stages, which could promote N-availability. TP in paddy water of BCF was most correlated to the bacteria, while COD was the most critical regulator for the fungi. Furthermore, correlation network analysis showed nutrient-cycling-related microorganisms were more closely interconnected in BCF than those in CF. These findings showed the application of biogas slurry plus chemical fertilizer could regulate rhizosphere microorganisms towards a beneficial fertilizer use for rice growth.

## 1. Introduction

Over the last decade, an increasing number of biogas plants have been built to tackle excessive manure from industrial livestock and poultry farms in China and other countries [1,2,3]. Biogas slurry, a by-product of biogas plants, is rich in organic matter, nutrients, and some bioactive substances, and also contains large amounts of water, which can meet the needs of paddy fields both for organic fertilizer and irrigation water [4,5]. Biogas slurry combined with chemical fertilizer (BCF) is commonly used as a fertilizer in actual paddy fields [6,7].

Additionally, rhizosphere microorganisms, which live in the narrow region of roots and interact directly with plants, play critical roles in the growth and reproduction of the agro-ecosystem [8] because they protect plants from adverse effects of abiotic stresses and facilitate plant growth [9]. As such, the responses of the rhizosphere microorganisms have been used as a valid indicator of crop management strategies including rotation regimes [10], fertilizer inputs [11], and cultivation patterns [12] in paddy fields. Some studies have been conducted to explore the effects of biogas slurry application on bulk soil microbial communities at a certain time [7,13,14]. As a matter of fact, the combination of biogas slurry with chemical fertilizer (BCF) is commonly applied in actual agriculture practice [15,16]. Moreover, the practical application of BCF is performed in phases, and the dynamic changes in rhizosphere microorganisms in a round of agricultural rotation deserve attention. However, few studies have been conducted to explore how the rhizosphere microorganisms change throughout the full life-cycle growth of rice with the application of biogas slurry plus chemical fertilizer. Therefore, it is essential to identify the dynamic changes in rhizosphere microorganisms during the whole life cycle of rice growth with the application of BCF to better guide the healthy production of crops and promote the effective use of biogas slurry in paddy fields.

Unlike other crops, rice is mostly cultivated in flooded paddy soils. The paddy water quality and depth often fluctuate with fertilizer inputs, rainfall, and irrigation, which alter the living conditions of rhizosphere microorganisms during rice growth [10]. However, it remains unknown to what extent the paddy water regulates the rhizosphere microorganisms, especially in the context of biogas slurry fertilization. It is thus necessary to fill the gap to ascertain the impacts of changing paddy water on rhizosphere microorganisms throughout the growth stages of rice with the addition of biogas slurry plus chemical fertilizer to paddy fields. 

In this work, two aspects were mainly taken into account. First, the dynamic responses of field-grown rice rhizosphere bacteria and fungi to biogas slurry plus chemical fertilizer were systematically investigated during the full life-cycle growth of rice. Second, the underlying relationship between paddy water properties and rhizosphere microorganisms in paddy fields was also explored. This research was of great significance for improving the understanding of the dynamic response of rhizosphere microorganisms of field-grown rice to biogas slurry plus chemical fertilizer and providing information about the proper fertilization regime in paddy fields.

## 2. Materials and Methods

### 2.1. Site Description and Crop Management

Two proximate paddy fields in Chongming Island, Shanghai, China were selected as field research sites considering their consistent crop management practices, including rice variety, seedling time, and fertilization time. One paddy field (named Beiyan) has been treated with biogas slurry plus chemical fertilizer for 4 years, while the other paddy field (named Xinsha) has been treated with chemical fertilizer. The amount of chemical fertilizer used in the Beiyan field was about one-third that of Xinsha. Table 1 shows the main properties of the soils under two different treatments. The biogas slurry was from the black film biogas digester tank in Shanghai Mingjin Animal Husbandry Co. Ltd., Shanghai, China. Before being applied to farms, the biogas slurry was mixed with river water in a ratio of 20 to 100 as basic fertilizer (application in early May before the rice seedling stage) and top-dressing fertilizer (application in June at the rice tillering stage). The amount of pure biogas slurry was 100 t/ha each time and the main properties of the biogas slurry were total nitrogen of 754–916 mg L^−1^ and total phosphorus of 112–300 mg L^−1^. The chemical fertilizer consisted of NPK compound fertilizer (N/P_2_O_5_/K_2_O = 15:15:15) and urea (46% N). Rice seedlings were planted in mid-May and the crop was harvested in mid-late September.

### 2.2. Sample Collection

The soil and paddy water were sampled throughout the rice growth stages, including seedling (19 d), tillering (45 d, 51 d, 68 d), booting (85 d, 99 d, 113 d), and grain-filling stages (130 d). Three replicates of plots of about 15 m^2^ were set up at each research field. Rhizosphere soils were collected using sterile brushes to brush off soils closely attached to the roots after uprooting the whole plants from each plot and shaking plants to separate the root systems from bulk soil. Paddy water samples were collected using sampling spoons to avoid disturbance. Considering the spatial heterogeneity of field rice paddies, five root soil samples (~100 g each) and five paddy water samples (~200 mL) in each plot were separately collected and then all the subsamples were mixed thoroughly as one representative testing sample. Each testing sample in each research field was taken in triplicate.

Rhizosphere soils and paddy water samples were immediately transported to the laboratory using cooler boxes for storage after mixing. After removing fine roots and small stones, the soil samples were divided into two parts. One was stored at −80 °C for the rhizosphere microorganisms community analysis, while the other was air dried for measuring soil chemical properties. Water samples were determined within 12 h.

### 2.3. Analytical Methods

The total nitrogen (TN) and total phosphorus (TP) of soil were determined by the Kjeldahl method and the sulfuric perchloric acid digestion method. The total organic carbon of soil (SOC) was measured using a TOC Analyzer (multi N/C 3100, Berlin, Germany). The concentrations of nitrite and nitrate in water samples were measured using ion-exchange chromatography (ICS-600, Thermo-Fisher, Shanghai, China) with an IonPac AS19 anion column. The concentrations of total nitrogen (TN), total phosphorus (TP), ammonium nitrogen (NH_4_^+^-N), and chemical oxygen demand (COD) of water samples were assessed according to the standard methods [17].

### 2.4. Microbial Characteristics Analysis

Soil samples for microbial analyses were gathered on day 19, 45, 85 and 130, and soil DNA was extracted from 0.5 g fresh soil using an E.Z.N.A. soil DNA Kit (Omega Bio-tek, Norcross, GA, USA) according to the manufacturer’s instructions. The bacterial and fungal community compositions were separately amplified using universal primers 515F (GTGCCAGCMGCCGCGG)/-806R(GGACTACHVGGGTWTCTAAT) [18] and ITS1F(CTTGGTCATTTAGAGGAAGTAA)/ITS2R(GCTGCGTTCTTCATCGATGC) [11]. Subsequently, genomic high-throughput sequencing was conducted using an Illumina MiSeq PE300 platform (Majorbio company, Shanghai, China). The raw sequence data have been deposited into the NCBI Sequence Read Archive database with the accession number PRJNA749128.

Questionable and chimeric sequences were examined and deleted using FLASH (v1.2.11), and qualified sequences with similarity > 97% were clustered into one operational taxonomic unit with Usearch (version 7.1). The numbers of 16S rDNA sequences from each sample of bacteria and fungi were separately rarefied to 33,203 and 50,224, which yielded an average Good’s coverage of 97.54% and 99.88%, respectively. The most abundant sequence for each OTU was selected as a representative sequence. Representative sequences from each OTU were identified using RDP Classifier (http://sourceforge.net/projects/rdpclassifier/ (accessed on 1 December 2021)). These analyses were performed on the free online platform of the Majorbio Cloud Platform (http://cloud.majorbio.com/ (accessed on 1 December 2021)).

### 2.5. Statistical Analysis

Fisher’s exact test was used to assess the statistical differences in the community structure of rhizosphere microorganisms during the rice growth between CF and BCF treatments, and *p*-values less than 0.001 were considered significant. One-way ANOVA and two one-sided equivalence tests were separately used to analyze the differences of soil data of CF and BCF treatments as well as the microbial community from triplicate testing samples. These aforementioned analyses were performed in SPSS 23.0 (IBM, USA). The co-occurrence pattern of predominant bacterial and fungal genera in four growth stages of rice in two treatments was constructed based on Spearman correlations, respectively. Co-occurrence events were identified with statistically robust correlations (|correlation coefficient| > 0.8 with *p*-value < 0.05) [19]. The obtained networks were visualized in the Gephi platform. In addition, correlations between microbial community structures and environmental variables were also performed by redundancy analysis (CANOCO 5.0).

## 3. Results and Discussion

### 3.1. Changes in Main Nutrients in Paddy Water during the Whole Growth of Rice

**Figure 1:**
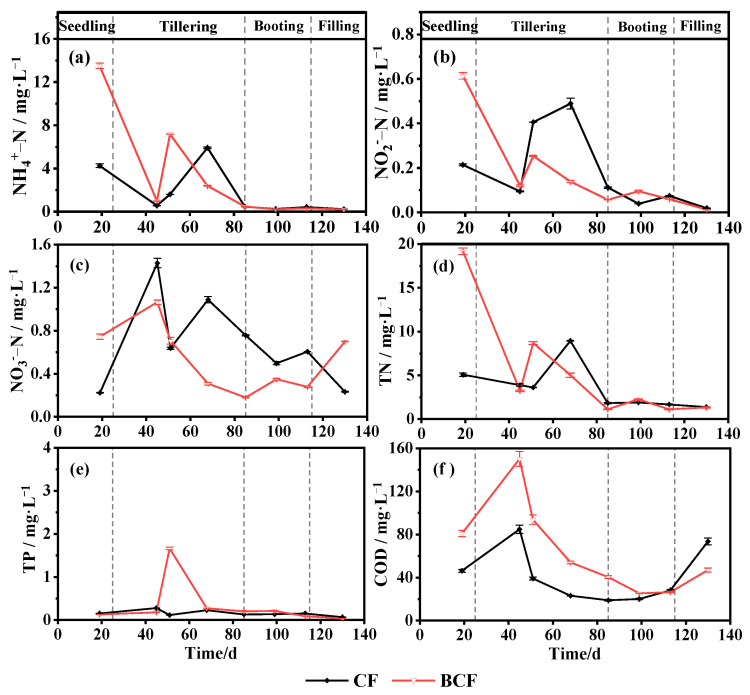
Changes in nutrient concentration in paddy water during the whole growth of rice in CF and BCF treatments. CF, chemical fertilizer, black line; BCF, biogas slurry plus partial chemical fertilizer, red line. (**a**) ammonium-N, (**b**) nitrite-N, (**c**) nitrate-N, (**d**) TN, (**e**)TP, (**f**) COD.

The whole rice growth cycle/process was divided into seedling (1–25 d), tillering (26–85 d), booting (86–115 d), and grain-filling (116–135 d) stages. Figure 1 shows the profiles of TN, ammonium-N, nitrite-N, nitrate-N, COD, and TP of the paddy water in the CF (chemical fertilizer treatment) field and BCF (biogas slurry plus chemical fertilizer treatment) field. In general, the concentrations of N- and P- nutrients and COD in the paddy water decreased during the rice growth for both CF and BCF fields. These observations were due to the persistent fertilizer consumption during the rice growth. 

Comparatively, the concentrations of TN, ammonium, and COD in BCF were much higher than those in CF, especially at vegetative stages (the seedling and tillering stages), with increases of 48.02%, 93.87%, and 55.23%, respectively. However, nitrate exhibited the opposite tendency. The mean nitrate level in BCF was 20.59% lower than that in CF. Additionally, the TP concentration in BCF increased sharply at the tillering stage after BS application and it was 118.75% higher than that in CF. The relatively higher contents of nutrients in BCF could be attributed to the high availability of liquid fertilizer (biogas slurry).

### 3.2. Changes in Microbial Diversity and Richness during the Whole Growth of Rice

**Figure 2:**
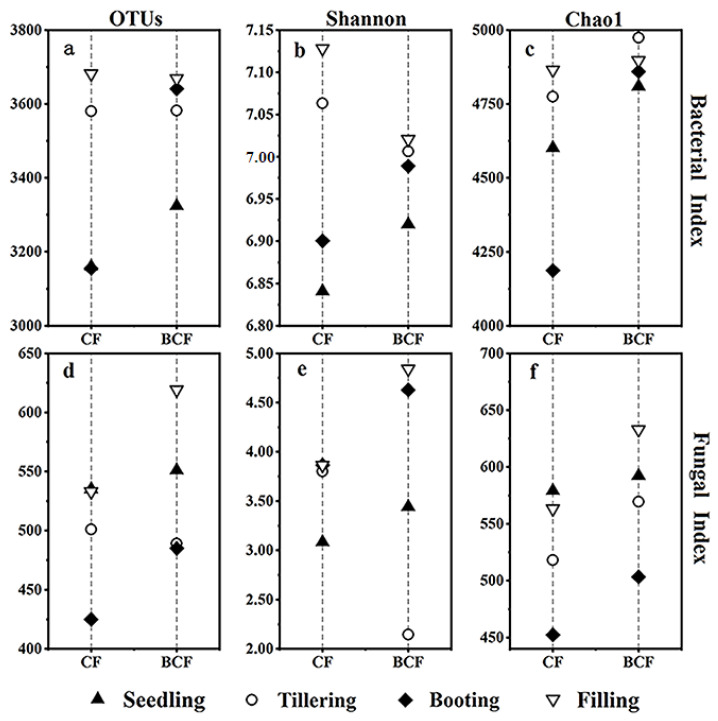
Diversity of the bacterial and fungal communities including OTU number (**a**,**d**), Shannon value (**b**,**e**), and Chao1 value (**c**,**f**) during the whole growth of rice in CF and BCF treatments. CF, chemical fertilizer treatment; BCF, biogas slurry plus partial chemical fertilizer treatment.

The changes in bacterial and fungal diversity and richness are displayed in Figure 2. The Shannon indices of bacteria and fungi both increased gradually in BCF during rice growth (Figure 2b,e). It was worth noting that the fungal Shannon indices in BCF were higher than those in CF, particularly in the booting and grain-filling stages. This finding showed that the application of biogas slurry combined with chemical fertilizer facilitated the fungal diversity. Additionally, the Chao1 indices of bacteria and fungi in BCF were both higher than those in CF at every growth stage of rice. The Chao1 index of rhizosphere bacteria increased generally with the growth of rice, whereas the opposite trend was observed in the fungi in both CF and BCF (Figure 2c,f). Generally, the microbial diversity and abundance in BCF were higher than those of CF.

### 3.3. Changes in Bacterial Community Structure during the Whole Growth of Rice

**Figure 3:**
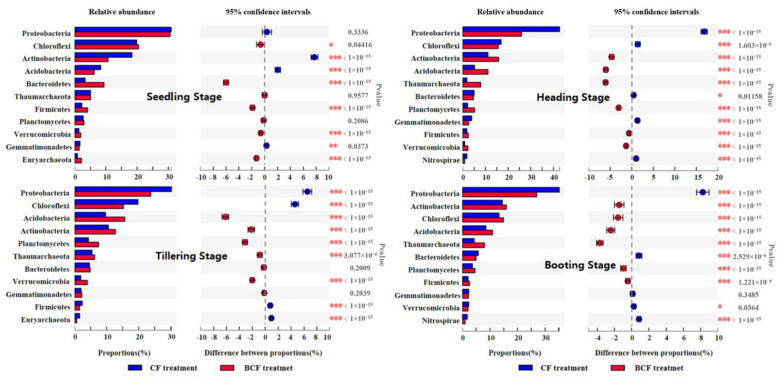
Rhizosphere microbial community structure during the whole growth of rice in CF and BCF treatments. Phyla differences in bacterial composition between these two treatments. Phyla proportions in one treatment have a positive (negative) difference with the other and are indicated by different colors. * *p* ≤ 0.05, ** *p* ≤ 0.01,*** *p* ≤ 0.001.

The taxonomic classification of the bacterial community revealed that Proteobacteria, Chloroflexi, Acitinobacteria, and Acidobacteria were the most frequently detected phyla in both CF and BCF (Figure 3). Differently, all the relative abundances of these dominant phyla in BCF were higher than those in CF. For example, the relative abundances of Proteobacteria and Chloroflexi in BCF (23.62–30.64% and 30.26–42.86%) were two to three times as much as those in CF (10.70–15.93% and 10.65–18.31%). It was noted that the relative abundances of Actinobacteria presented increasing trends with the growth of rice in BCF and CF, and a marked fluctuation of the relative abundances of Acidobacteria was observed during rice growth. Additionally, bacterial communities from triplicate testing samples were generally equivalent (*p* < 0.05).

It was also found that some genera related to nitrogen (N), phosphorus (P), and carbon (C) transformation were more abundant in the rice root zone in BCF than in CF (Table 2). Notably, the relative abundances of AOA (Ammonia-Oxidizing Archaea)-related genus were higher than those of AOB (Ammonia-Oxidizing Bacteria) in both CF and BCF. The predominant AOA-related genus was *Nitrososphaeraceae* (CF, 1.51–4.26% and BF, 4.04–6.43%), which suggested that *Nitrososphaeraceae*-like AOA played a critical role in the ammonium oxidation process. Moreover, the relative abundance of *Nitrososphaeraceae*-like AOA demonstrated a growing trend in BCF during the whole growth of rice. Additionally, the relative abundances of the dominant denitrifying bacteria (i.e., *Steroidobacteraceae*) decreased in BCF and increased in CF during rice growth.

Differently, the relative abundances of DNRA (Dissimilatory Nitrate Reduction to Ammonium)-related bacteria (e.g., *Geobacter*, *Anaeromyxobacter*, and *Ignavibacterium*) in BCF presented a growing tendency at the reproductive stages (i.e., booting and filling). These observations showed BCF treatment could reduce the N-loss pathway and promote N-availability during the whole growth of rice. In addition, phosphate-solubilizing bacteria (e.g., *Nitrososphaeraceae*, *Streptomyces*, *Bacillus*, and *Rhodococcus*) and organic matter degraders (e.g., *Anaerolinea* and *Marmoricola*) were more abundant in BCF than in CF at each rice growth stage, and their relative abundances increased in BCF and fluctuated in CF during rice growth.

**Figure 4:**
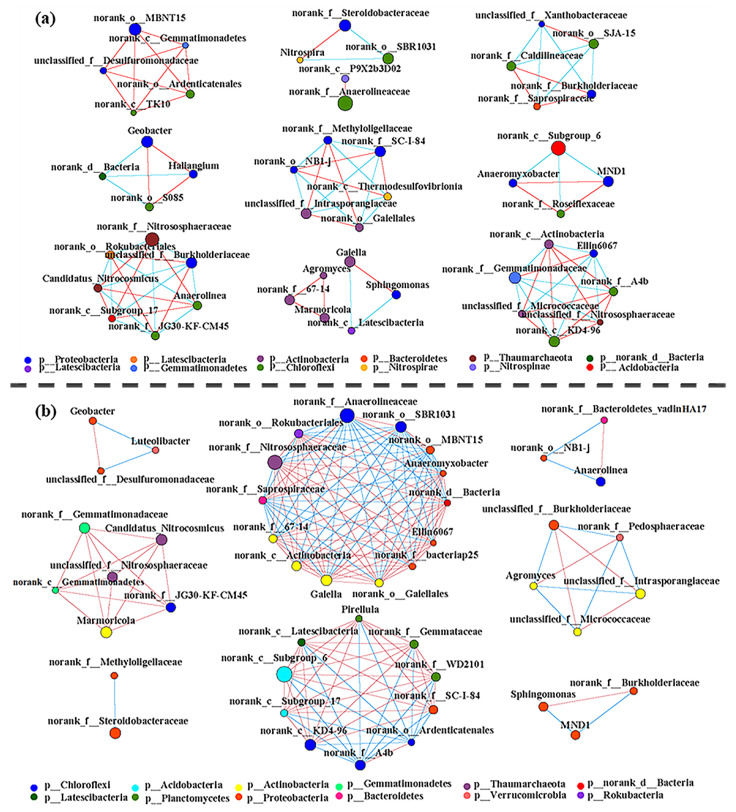
Correlation network of bacteria taxa at genus level: (**a**) CF, chemical fertilizer, (**b**) BCF, biogas slurry plus small amount of chemical fertilizer. Each dot refers to a bacterial phylotype (an OTU clustered at 97%). Connections represent significant (*p* < 0.01) correlation, blue and red links represent positive and negative correlations between genera, respectively. The size of each node is proportional to genera richness.

Additionally, correlation analysis of the predominant genera was also performed at the four rice growth stages of BCF (Figure 4a) and CF (Figure 4b) to ascertain their coupling relationship. The co-occurrence networks in the BCF and CF treatments consisted of 88 nodes with 339 edges and 94 nodes with 183 edges, respectively (Figure 4). Comparatively, the bacterial network in BCF treatment was more complex and showed a closer correlation of N-, P-, and C-cycling-related microbes. In particular, the dominant Proteobacteria, Actinobacteria, *Thaumarchaeota*, and Ascomycota were highly positively correlated in BCF treatment, as evidenced by the intertwined red wide connection lines (Figure 4b). It was noted that organic matter degradation-related *Marmoricola* and *Gaiella* [8] were correlated with the N-cycling-related genus *Nitrososphaeraceae* [20] in BCF.

### 3.4. Changes in Fungal Community Structure during the Whole Growth of Rice

**Figure 5:**
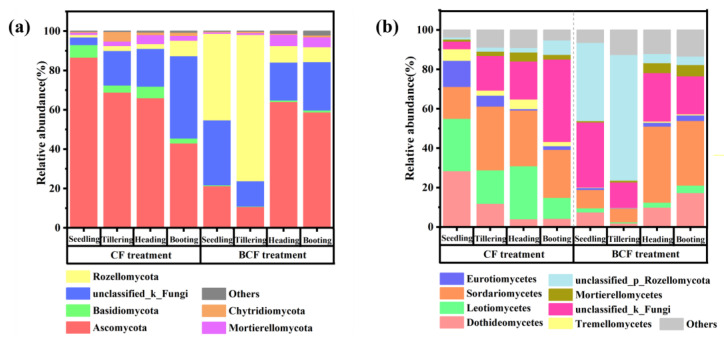
(**a**) The relative abundance of the dominant fungi at phylum level. (**b**) The relative abundance of the dominant fungi at class level. CF, chemical fertilizer treatment; BCF, biogas slurry plus partial chemical fertilizer treatment.

The main phyla of fungi in CF and BCF were Ascomycota, Basidiomycota, Rozellomycota, Mortierellomycota, and Chytridiomycota (Figure 5a). Comparatively, the relative abundance of the predominated fungal phylum was Ascomycota in CF, which decreased from 86.70% to 43.09% during rice growth. However, Ascomycota was only dominant in the booting stage (64.14%) and grain-filling stage (58.86%) in BCF, and their corresponding relative abundances of Ascomycota were higher than those in CF. The Ascomycota-like *Dothideomycetes* and *Sordariomycetes* were found to be the dominant fungi class at booting and filling stages in BCF (Figure 5b).

The relative abundances of fungal genera related to nutrient transformations in CF and BCF are also presented in Table 2. It was worth mentioning that *Mortierlla* played crucial roles in decomposing plant litter and solubilizing soil phosphate [21], and were more abundant at booting and grain-filling stages than seeding and tillering stages for both treatments. In particular, Ascomycota was found to be the critical phylum that was linked to other fungal communities.

### 3.5. Relationships between the Rhizosphere Microbial Communities and Environmental Factors

**Figure 6:**
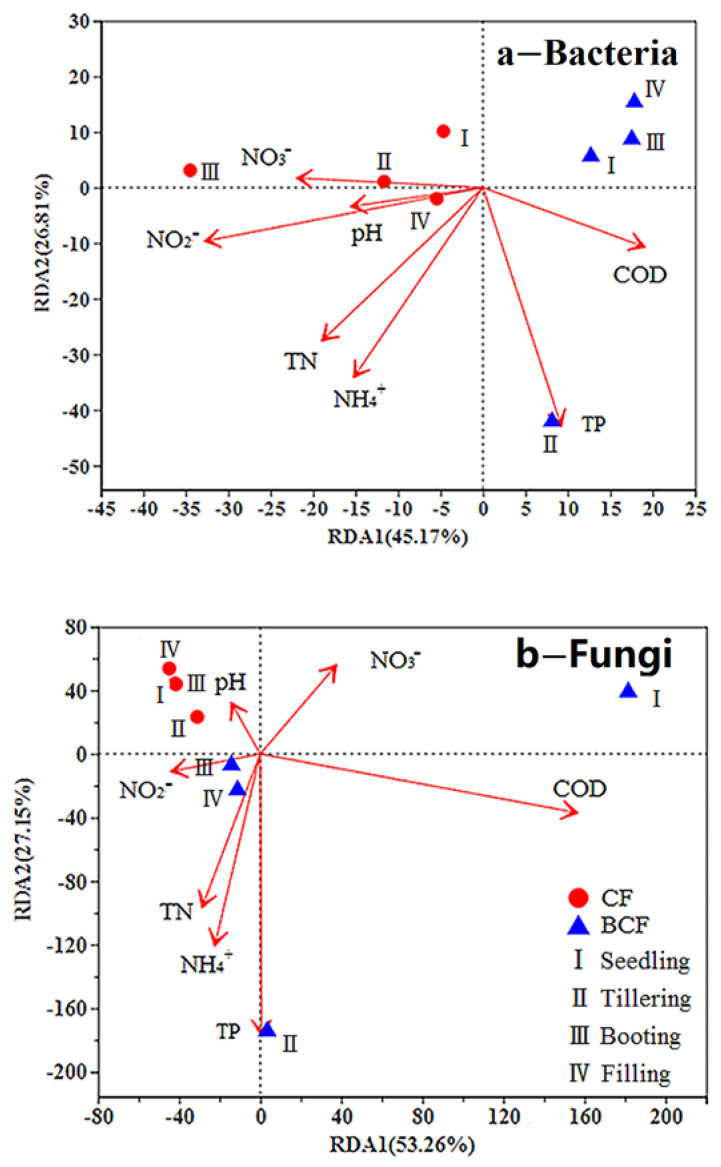
Redundancy analysis (RDA) ordination plots depicting the relationships between rhizosphere bacterial (**a**) and fungal (**b**) communities and paddy water properties. TN, total nitrogen; TP, total phosphorus; NH_4_^+^, ammonium nitrogen; NO_2_^−^, nitrite nitrogen; NO_3_^−^, nitrate nitrogen; COD, chemical oxygen demand; CF, chemical fertilizer treatment; BCF, biogas slurry plus partial chemical fertilizer treatment.

The redundancy analysis (RDA) was performed to reveal the relationships of dominant bacterial and fungal community compositions with the environmental factors (TN, NH_4_^+^, NO_2_^−^, NO_3_^−^, TP, COD, pH of paddy water) (Figure 6). The RDA profile showed that the first two axes explained 71.98% and 80.41% of the total variations of the bacterial and fungal community differences, respectively. It was found that the bacteria in the BCF were negatively correlated with NO_3_^−^ at different growth stages and positively correlated with TP at the tillering stage, whereas the bacteria in the CF were positively correlated with NO_3_^−^ and negatively correlated with TP at different growth stages. It was worth noting that TP in the paddy water of BCF was most correlated to the rhizosphere bacterial community structures (R^2^ = 0.8946, *p <* 0.05), while COD in the paddy water was the most important factor in shaping the fungal community (R^2^ = 0.8794, *p <* 0.05).

### 3.6. Discussion

Rhizosphere microbial communities play an important part in stimulating the fertilizer utilization in the soil and further affecting plant growth [22,23,24]. In comparison with chemical fertilizer, biogas slurry fertilizer plus chemical fertilizer can quickly increase the contents of nutrients (e.g., ammonium and TP) in paddy water and is also rich in organic matter, which can regulate rhizosphere microbial communities. The relatively high abundance of denitrifying bacteria at the vegetative stages (seeding and tillering) in BCF can be explained by the fact that excessively higher organic matter from biogas slurry creates a more favorable micro-environment for denitrification due to the depletion of oxygen and the easy availability of carbon sources. Although some work shows high-level organic matter is favorable for DNRA [25], the abundances of DNRA-related bacteria are lower than those of denitrifying bacteria in BCF. The relatively high free ammonium (about 13.51 mg L^−1^) inhibiting DNRA in BCF may explain the observation. A similar finding has been reported that NH_4_^+^-rich circumstances suppress the DNRA process [26]. Additionally, the type of carbon and nitrogen sources and pH are also key in regulating denitrification and DNRA [27,28].

It is worth noting that the relative abundance of DNRA-like bacteria increases and that of denitrifying bacteria decreases during the rice growth in BCF, which helps to reduce N loss and promote N retention. In addition, the relatively higher abundances of phosphorus-solubilizing bacteria (PSB) and organic matter degraders in BCF than those in CF showed that the application of BS can facilitate nutrient availability and soil fertility in BCF. Importantly, the gradual increase in PSB and organic matter degraders during rice growth can also facilitate nutrient utilization at low fertilizer input stages. Taken together, these findings suggest the application of BCF may create a preferable microenvironment for key rhizosphere microorganisms favoring rice cultivation.

As another indispensable component of rhizosphere microorganisms, fungi are equally important in promoting the rhizosphere nutrient cycling [29]. Compared with bacteria, fungi have a stronger ability to degrade recalcitrant litter [30]. For example, the dominant Ascomycota-like fungi (e.g., *Sordariomycetes* and *Dothideomycetes*) are mainly responsible for lignocellulose decomposition [31,32]. In addition, the abundances of Ascomycota presented the trend of decrease at the first two stages and then increased at later stages in BCF, which is different from the report that Ascomycota numbers increase in response to biogas slurry application [33]. This difference may be owing to the previous work only focusing on a certain stage rather than the whole rice growth stages. Considering Ascomycota are vulnerable to high N, P, and C inputs [34], the higher nutrient contents in paddy water at early stages might exert pressure on the growth of Ascomycota in BCF. After an adaptive response to higher levels of nutrients, Ascomycota begins to increase at later stages. The relatively higher abundance of Ascomycota at booting and filling stages shows soil organic matter decomposition may be accelerated at the key reproductive stages in BCF and thus contribute to increasing available nutrients. Hence, the higher abundance of Ascomycota at grain-limiting reproductive stages may be important to rice growth.

Revealing the linkages between microorganism communities is crucial for the understanding of ecosystem functioning. The correlation network showed that the microbial community was more closely correlated in the BCF than in the CF. The closer linkage of N-, P-, and C-cycling-related bacteria and fungi in BCF indicated that the application of BS could increase the interconnection among the rhizosphere microorganisms, and thus result in better use of nutrients as well as improve the tolerance of plants against adverse environmental stress.

Additionally, there are different responses to environmental factors between the rhizosphere bacterial and fungi communities in BCF. Bacterial communities are mostly correlated with TP, while fungal communities are closely related to COD in BCF. This observation further proves the above deduction that fungi are mainly responsible for organic matter (e.g., lignocellulose) decomposition. Different from BCF, nitrate is the important influencing factor for bacterial communities in CF. The difference in nitrate nitrogen in paddy water of BCF and CF may lead to the discrepancy in the relative abundance of proteobacteria between the two treatments. High phosphorus input directly changes bacterial community structure in P-limited paddy soil [35]. The significantly higher content of TP at tillering stage of the BCF may account for the differences in bacterial community between the BCF and CF. Notably, there was a higher explanatory rate between TP and Acidobacteria. In sum, this research is of great significance for further elucidating the relationship between paddy water properties and the rhizosphere microbial community.

## 4. Conclusions

This study demonstrated that the application of biogas slurry plus chemical fertilizer in a paddy field was an effective practice by increasing N, P, and C contents, as well as rhizosphere microorganism abundance and diversity. Biogas slurry promoted rhizosphere bacteria and fungi to participate in N, P, and C cycling, as evidenced by higher abundances of phosphate-solubilizing and cellulose-decomposition-related bacteria (e.g., *Bacillus*) and fungi (e.g., *Mortierella*), as well as N-cycling-related bacteria (e.g., *Nitrososphaeraceae*) in the rice rhizosphere in BCF, especially at the booting and filling stages. It was worth noting that the relative abundance of DNRA-like bacteria increased and that of denitrifying bacteria decreased during rice growth, which could promote the retention of NH_4_^+^-N and nitrogenous nutrients’ availability at low fertility input stages in BCF. Additionally, bacterial communities are mostly correlated with TP, while fungal communities are closely related to COD in BCF. In summary, the application of biogas slurry plus chemical fertilizer could facilitate the nutrient-cycling-related rhizosphere microorganisms towards a beneficial fertilizer use in paddy fields.

## Figures and Tables

**Table 1:** Main properties of the soil under two different treatments.

Treatment	pH	TN (g kg^−1^)	TP (g kg^−1^)	TOC (g kg^−1^)	AN (mg kg^−1^)	AP (mg kg^−1^)	AK (mg kg^−1^)
CF	7.48 ± 0.04 a	2.62 ± 0.10 a	0.75 ± 0.03 a	15.69 ± 3.20 a	0.16 ± 0.08 a	40.50 ± 5.25 a	236.36 ± 6.43 a
BCF	7.50 ± 0.05 a	2.72 ± 0.20 a	0.79 ± 0.05 a	17.29 ± 2.91 a	0.16 ± 0.04 a	43.90 ± 6.11 a	220.76 ± 10.55 a

Notes: All the values are presented as mean ± SD (*n* = 3), the same letter indicates no significant difference between treatments by one-way ANOVAs (*p >* 0.05). TN, total nitrogen; TP, total phosphorus; TOC, total organic carbon; AN, available nitrogen; AP, available phosphorus; AK, available potassium; CF, chemical fertilizer treatment; BCF, biogas slurry plus partial chemical fertilizer treatment.

**Table 2:** Dynamic changes in abundances of nitrogen-, phosphorus-, and carbon-cycle-related microbial communities under different treatments (%).

	Microbes	Genus/Family	CF				BCF			
			Seedling	Tillering	Booting	Filling	Seedling	Tillering	Booting	Filling
N-cycle- related microbes	AOB	*Nitrosospira*	0.05	0.02	0.09	0.01	0.12	0.07	0.03	0.06
		*Nitrosomonas*	0.01	0.04	0.02	0.06	0.04	0.05	0.02	0.04
	AOA	*Nitrososphaeraceae*	3.99	4.26	1.51	3.48	4.04	5.05	6.09	6.43
		*Ca.Nitrocosmicus*	0.92	1.14	0.37	0.62	1.21	1.20	1.82	1.45
	NOB	*Nitrospira*	0.32	0.42	0.63	0.78	0.25	0.36	0.61	0.50
	Denitrifying bacteria	*Steroidobacteraceae*	1.32	1.63	1.69	1.93	1.67	1.85	1.30	1.15
		*Thiobacillus*	0.76	0.49	0.50	0.09	1.23	0.17	0.12	0.04
		*Pseudomonas*	0.78	0.30	0.23	0.27	1.00	0.22	0.10	0.11
		*Rhodobacter*	0.07	0.1	0.11	0.39	0.45	0.31	0.13	0.05
		*Steroidobacter*	0.14	0.10	0.15	0.23	0.10	0.20	0.24	0.35
		*Thauera*	0.07	0.13	0.24	0.20	0.36	0.15	0.12	0.11
	DNRA bacteria	*Geobacter*	1.39	1.70	1.87	1.29	0.71	0.42	0.43	0.92
		*Anaeromyxobacter*	0.62	0.56	1.32	0.58	0.34	0.38	0.51	0.72
		*Ignavibacterium*	0.18	0.21	0.31	0.10	0.10	0.08	0.10	0.11
	AnAOB	*Ca.Brocadia*	-	0.003	0.003	0.007	0.007	0.008	0.010	0.013
		*Ca.Anammoximicrobium*	0.013	0.003	-	0.005	0.051	0.022	0.01	0.013
Phosphate- solubilizing- related microbes	PSB	*Nitrososphaeraceae*	3.99	4.26	1.51	3.48	4.04	5.05	6.09	6.43
		*Streptomyces*	0.47	0.20	0.21	0.24	0.39	0.34	0.39	0.46
		*Bacillus*	0.39	0.36	0.30	0.34	0.49	0.23	0.58	0.51
		*Rhodococcus*	0.09	0.07	0.07	0.24	0.13	0.09	0.13	0.14
	Fungi	*Aspergillus*	10.30	5.14	0.22	0.96	0.70	0.04	0.33	0. 47
		*Mortierella*	1.06	2.17	4.62	2.38	0.70	0. 92	5.74	5.03
Organic- matter- degrading- related microbes	Bacteria	*Burkholderiaceae*	0.83	0.78	2.06	1.64	1.29	0.83	1.05	0.99
		*Bacillus*	0.39	0.36	0.30	0.34	0.49	0.23	0.58	0.51
		*Gaiella*	1.32	0.98	1.26	1.61	0.48	1.36	1.56	1.77
		*Marmoricola*	1.31	0.86	0.88	1.02	1.10	1.20	1.83	1.57
		*Anaerolinea*	1.09	1.11	0.46	0.60	1.51	0.79	0.57	0.84
	Fungi	*Pyrenochaetopsis*	27.25	10.16	2.71	3.27	5.45	1.59	14.26	6.48
		*Mortierella*	1.06	2.17	4.62	2.38	0.70	0.92	5.74	5.03
		*Penicillium*	0.04	0.03	0.07	0.10	0.03	0.04	0.40	0.62
		*Acremonium*	0. 576	0.244	0.003	0. 259	0.232	0.070	0.214	0.550
		*Gibberella*	0. 27	0. 33	0.07	0.01	0.18	0. 53	3.88	2.57
		*Hydnodontaceae*	-	-	-	-	0.006	-	-	-

Notes: AOB, ammonia-oxidizing bacteria; AOA, ammonia-oxidizing archaea; NOB, nitrite-oxidizing bacteria; DNRA, dissimilatory nitrate reduction to ammonium; AnAOB, anammox bacteria; PSB, phosphate solubilizing bacteria; CF, chemical fertilizer treatment; BCF, biogas slurry plus partial chemical fertilizer treatments.

## Data Availability

The authors confirm that the data supporting the findings of this study are available within the article.
